# Integrated Care for People Living With Rare Disease: A Scoping Review on Primary Care Models in Organization for Economic Cooperation and Development Countries

**DOI:** 10.1177/21501319241311567

**Published:** 2025-01-08

**Authors:** Nada Vidic, Anna McGlynn, Fatemeh Abdi, Chun Wah Michael Tam, Reginald Michael Crampton, Kean-Seng Lim, Elizabeth Emma Palmer, Natalie Taylor, Ben Harris-Roxas

**Affiliations:** 1 University of New South Wales, Sydney, NSW, Australia; 2 Population and Community Health Directorate, Sydney, Australia; 3 Primary and Integrated Care Unit, Liverpool, NSW, Australia; 4 Rosedale Medical Practice, Sydney, NSW, Australia; 5 WentWest Primary Health Network, Sydney, NSW, Australia; 6 Mt Druitt Medical Practice, Sydney, NSW, Australia; 7 University of Technology Sydney, Sydney, NSW, Australia

**Keywords:** general practitioners, family physicians, implementation science, delivery of care, integrated, continuity of patient care

## Abstract

**Introduction/Objectives:**

Individually rare, rare diseases are collectively common resulting in frequent health system use. Navigating the health system persists as a challenge. Primary care provides longitudinal contact with the health system and is placed to provide integrated rare-disease-care.

**Methods:**

This scoping review used Joanna Briggs Institute and PRISMA methods with a Consolidated Framework for Implementation Research based data extraction tool to find how integrated rare-disease-care is delivered, enablers and barriers to the same, in primary care settings in contemporary literature in OECD countries.

**Results:**

The Primary Care Provider (PCP) role varies from routine primary care to shared-rare-disease-care models. In the 26 papers, the most frequently cited PCP roles included involvement in diagnosis (n = 14), care coordination (n = 16), primary and preventative care (n = 18), management of components of rare-disease-care (n = 13), and treatment monitoring (n = 10). Individuals whose PCP was actively involved in their care were reported to have shortened diagnostic delay, improved transitions of care across the lifespan, reduced unplanned utilization of emergency and hospital services, comprehensive psychosocial care, improved quality of life across environments including home, school and work and improved palliative care experiences.

**Conclusions:**

Sufficient communication from specialists, information, resources, time and reimbursement for complex care are still needed. Future integrated-rare-disease-care models should be developed by, or with, PCPs.

## Introduction

A disease is considered rare when the population prevalence is less than 5 in 10 000 people.^1^ There are over 9000 individual rare diseases, examples include cystic fibrosis, scleroderma, and epidermolysis bullosa.^2,3^ Rare diseases are generally chronic, complex, progressive and may be life-limiting.^1,4,5^ An estimated 95% of rare diseases do not have a prognosis-altering therapy.^6,7^ Approximately 80% start exclusively in childhood and 30% of this group die before the age of 5 years.^8^ Of all rare diseases, it is estimated that over 70% have a genetic origin.^6^ Up to half of people living with a rare disease do not have an identified diagnosis.^6,9^ In these situations, the term “undiagnosed rare disease” is used. This may be due to challenges identifying that the person has an underlying rare disease, or due to challenges confirming an underlying etiology. The rare disease literature commonly refers to a person undergoing a “diagnostic odyssey,” a term used to describe the extended time spent attending multiple health providers, undertaking tests and procedures to confirm a diagnosis: the average diagnostic delay is 5 to 7 years, and 4 out of 10 people living with a rare disease in Australia reporting seeing more than 6 doctors and having at least 1 misdiagnosis^9,10^ This article acknowledges that rare disease impacts families, caregivers and support people, for brevity the term People Living with Rare Disease (PLWRD) is used to refer to this whole group.

Internationally, PLWRD report similar challenges with gaining a diagnosis, care coordination and access to information and support regardless of their specific diagnosis.^11,12^ Health professionals caring for PLWRD are presented with challenges associated with infrequent presentations of individual rare diseases.^13^ PLWRD may present with signs and symptoms that masquerade as common diseases, may be overlapping and non-specific and/or not fully observable in younger individuals.^14^ Tertiary/quaternary, care providers, termed “specialists” throughout, may only encounter 1 or 2 individuals with the same rare disease in their career, even in large tertiary centers.^15^ Yet, Primary Care Physicians (PCPs) are likely to frequently encounter a range of distinct rare diseases. In the Australian context, each full-time equivalent PCP has an average of 72 individuals with a rare disease “on their books,” diagnosed or with an “undiagnosed rare disease.”^16,17^

As the first contact with a health system, primary care consists of PCPs, nurses and allied health professionals, ideally working in multidisciplinary fashion to provide comprehensive, continuous, and coordinated collaborative care in a holistic, person-centered manner.^18,19^ PCPs are experienced in providing health education, bio-psycho-social care, support, advocacy and coordination between other sectors such as hospitals and social and welfare organizations.^18,20^ PCPs are familiar with dealing with diagnostic uncertainties and are ideally positioned to provide integrated-rare-disease-care for family units, they generally have life-long relationship with patients, often from multiple generations.^19^

Increased international rare disease research collaboration and innovations such as genomics, have resulted in increasing life expectancy of PLWRD. Improved delivery and coordination of care is an emerging need.^14,20-26^ How care is organized and delivered between multidisciplinary providers is termed as “integrated-rare-disease-models-of-care” for the purposes of this paper.

Integrated-rare-disease-models-of-care are important to bridge current health equity gaps and are a public health priority.^27^ However, their development presents challenges that are unique from other chronic, but common, diseases. Low prevalence results in a lack of research and clinical guidelines. Compounding this are the diversity and multitude of disease entities, their chronic, and often disabling nature, heterogenous manifestations, multi-system involvement, and diagnostic and treatment complexity. Avoidable gaps in multidisciplinary service delivery are the very reasons integrated-rare-disease-care is needed.^23,28,29^ Currently, the weight of coordinating and communicating between the multitude of services and care providers most commonly sits with PLWRD.^28,30,31^

Anticipating the key success factors and understanding PCP perspectives on how integrated-rare-disease-care is organized and delivered before new approaches are introduced is important and timely.^32^ The Consolidated Framework for Implementation Research (CFIR 2.0) supports systematic categorization of the innovation type (integrated-rare-disease-models-of-care), the inner setting (the primary care practice), outer settings (primary healthcare and wider health and social systems), the characteristics of the individual delivering the care (the PCP), and the implementation process, if reported.^33,34^ Standardized, clear descriptions of components of integrated-rare-disease-models-of-care are required to ensure success can be replicated and key problems mitigated across settings.^35^

This scoping review assesses the extent to which contemporary literature clarifies key characteristics, components or factors related to integrated-rare-disease-care delivery, along the rare disease patient journeys, from the perspective of primary care settings, in Organizations for Economic Cooperation Development (OECD) countries, to answer:

Primary question: What characteristics, components or factors related to integrated-rare-disease-models-of-care, in primary care settings, are described?
Secondary question: What are the reported outcomes of the same?
Tertiary question: What are the enablers and barriers to PCP involvement in integrated-rare-disease-care?

## Methods

The previously published scoping review protocol was guided by the methodological framework proposed by the Joanna Briggs Institute Manual for Evidence Synthesis.^36,37^ Microsoft Excel version 16.74 and Covidence (2023) were used to track, organize, and extract data.^38^ The protocol was refined through discussion with PCP advisors (CWMT, MRC, KSL) and the author expertise and experience in supporting PCPs (NV, AM), and primary care research (NV, BHR).

Eligible studies were published in English between 2013 and 2023, with no limitations on grey literature nor study design. Literature describing and/or testing at least one component of how rare-disease-care is organized or delivered, across care settings, from the perspective of primary care were the focus. The inclusion/exclusion criteria for eligible studies are summarized in Supplement I.

Study screening was completed by the main author (NV) and co-screened by (AM, FA) at 2 levels: (i) title/abstract and (ii) full text, with each reaching 80% agreement prior to progressing. Discrepancies regarding study relevance were resolved via group discussion. Full text papers were coded deductively by the first author (NV) using extraction (Supplement II), including relevant CFIR 2.0 domains (Supplement III).

A sample of 10% were checked by co-author (NT) relating to the CFIR 2.0 domains. One researcher (NV) coded all remaining components according to rules agreed during this discussion. Data analysis and interpretation were discussed with PCP advisors (CWMT, MRC, KSL) to gain industry knowledge and contextualize results.^39^

## Results

Using the search terms and snowballing techniques, 1318 papers were identified. Duplicate removal and screening yielded 169 full-text papers, which were assessed for eligibility. With 26 papers being included in the final review.

**Figure 1.**
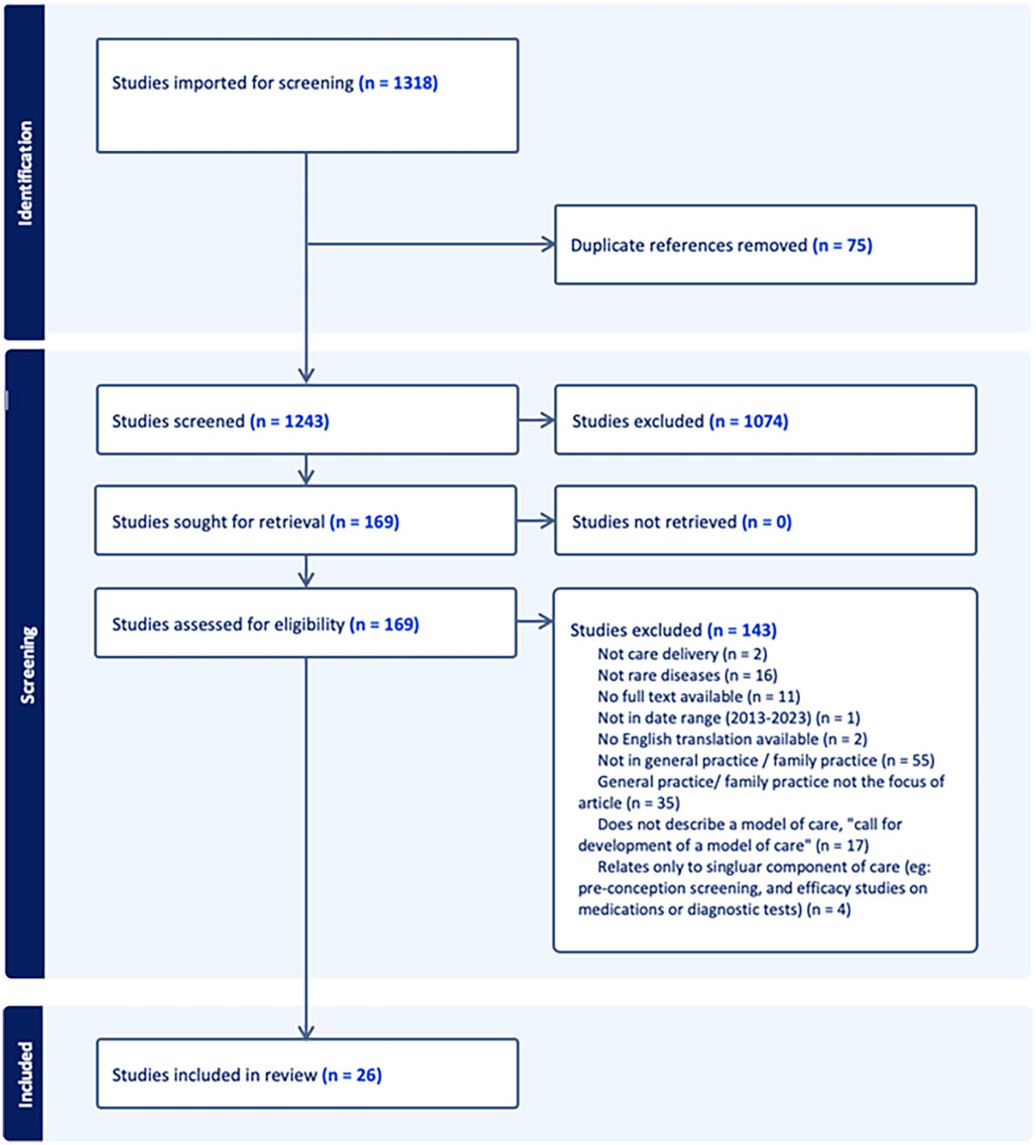
PRISMA scoping literature review flowchart.

At the full-text stage, categories of excluded papers were: where primary care settings were not the focus (n = 90); calls for a model of care to be developed, rather than describing or evaluating a model or component of integrated-rare-disease-care (n = 17); not related to a rare disease (n = 16), and those relating to a single clinical encounter or component of care, such as pre-conception screening (n = 4) were also excluded. Articles noting the importance of PCP’s role in the coordination and integration of rare-disease-care through brief statements such as “involve the patient’s PCP” were frequent (n = 22) yet excluded due to superficiality. Figure 1: PRISMA, provides further detail on the screening process.

### Key Characteristics, Components or Factors Related to Integrated-Rare-Disease-Models-of-Care, in Primary Care Settings

Descriptive characteristics of the studies are outlined in Supplement V: Descriptive Characteristics and Supplement VI: Study Characteristics: Disease, Context and Care Delivery. The study designs, aims and phenomena of interest are described in Supplement VII: Main characteristics of included studies.

Many included papers emphasized that coordination of services, including medical, clinical, and social and community services, are central to a multidisciplinary approach. In the 26 included papers, the most frequently cited components of care delivery by PCPs included: routine primary care management (n = 18), care coordination (n = 16), diagnosis (n = 14), management of components of rare-disease-care (n = 13) monitoring of rare disease treatment set by the specialist (n = 10).

Of all 1318 papers, 173 were concerned with transition of care from the pediatric to the adult health system, as a time where care coordination is particularly required. However, only 6 transition-related articles met all components of the eligibility criteria and were included. Components of care delivery in relation to PCP as described in the literature are defined in Table 1.

**Table 1.** Component of Care Delivery in Relation to the Role of the Primary Care Physician (PCP).

Component of care	PCP role	n	References
Prevention of *in-utero* exposure	Education	1	Original research^61^
Screening and diagnosis	Identification of potential rare disease	14	Original research^52,53,55,56,61,62,63^ Review^31^ Guidance document^45,58#,59 #^ Commentary, Opinion or Editorial^43 #,47,48^ Grey or Pre-print^60^
Management (Primary care)	Routine primary and preventative care delivery (immunizations, nutrition, hearing and vision screening, safety planning, pre-natal care, sexual health), education and management of concurrent illnesses	18	Original research^52-56,61,62^ Review^44,51^ Commentary, Opinion or Editorial^42,43 #,44,46-48,58 #^ Grey or Pre-print^50,60^
Management of components of the rare disease in primary care	Co-management, shared-care models, emergency management or management of rare disease complications, mild symptom management, advising of clinical trial opportunities, repeat prescriptions or blood test referrals	13	Original research^49,53,55-57^ Review^31,44,51^ Guidance document^44^ Commentary, Opinion or Editorial^43 #,46,48^ Grey or Pre-print^50^
Treatment monitoring	Monitoring of treatment initiated by specialist, encouraging medication and therapy adherence, specialist-only models	10	Original research^41,55,62^ Review^31,44,51^ Guidance document^44^
Care coordination	Navigating the health system, organization of care pathways, referrals, care plans, flow of medical information, mode of communication, communication between providers and to family	16	Original research^28,52-54,61,62^ Review^51^ Guidance document^44,45,58 #^ Commentary, Opinion or Editorial^43 #,46-48^ Grey or Pre-print^50,60^
Support	Psycho-social family-centric) mental health screening and management	6	Original research^52,62^ Review^44,51^ Guidance document^44,59 #^
Advisory and advocacy	Coordinating role for phyco-social, behavioral, patient advocacy, disability and educational sectors	6	Original research^61^ Review^31,44^ Guidance document^44,59 #^ Commentary, Opinion or Editorial^48^
Transition	Support and coordination of transitional care form pediatric to adult services, or across lifespan	6	Original research^57,61^ Review^44,51^ Guidance document^44^ Grey or Pre-print^60^
Palliative care	Support and coordination of services	1	Review^51^

# Investigates or proposes an ideal rare-disease-model-of-care delivery.

The roles of the PCP in how care is organized and delivered are summarized in Supplement VII: Models of Rare Disease Care. This supplement (VII) also groups enablers and barriers to active PCP involvement in rare-disease-care in each study mapped to the quadruple aim of health. Namely, patient outcomes, patient experience, provider experience and health system outcomes.^40^

PCP involvement in rare-disease-care is low in specialist-only rare-disease-care, where the PCP role focuses on providing primary care management.^41,42^ Shared-care models include those where decisions are made in consultation with PCPs, who assume responsibility of chronic care that are feasible in the primary care setting in addition to providing primary and preventative care.^31,43-52^ Papers investigating consultation length, encounter frequency and number of- and appropriateness of referrals, showed that PCPs are more actively involved in the care of PLWRD compared to general patient population, after controlling for patient characteristic (age, sex, insurance type, number of chronic diseases and whether they are an established or new patient) at the same practice, regardless of the model of care.^53,54^

### Outcomes of PCP Involvement in Rare-Disease-Care

Patient and provider level outcomes are both reported to be improved when PCPs have more active involvement in rare disease care.^28,55-57^ Reported outcomes of PCP involvement are in Table 2.

**Table 2.** Reported Outcomes of Rare-Disease-Care Delivery That Include Primary Care Settings.

Outcome domain	Reported outcomes	Reference
Patient health outcomes	Reduced diagnostic delay, high involvement in diagnosis	^31,44,53,55,59^
	Reduced disease activity	^31,43,57^,
	Increased lifespan	^45^
	Reduced unplanned emergency or hospital utilization	^31,43,49,60^
	Improved psychosocial support and quality of life	^28,31,43,44,52,59^
	Improved palliative care experience	^31^
	Improved care for broader health, as compared to specialist-only models for young adults	^51^
Patient experience outcomes	Improved with long-term (or lifelong) patient-PCP relationship	^46-49,51-53,58,60^
	Improved with multidisciplinary care	^31^
	Improved care coordination across the continuum and integration with social services	^31,44,45,46,51^
	If directed to primary care without coordinated transfer patient experience is reduced	^57^
	Family choice in care provider improved experience of care	^52,57^
Provider experience outcomes	Decreased with perceived insufficient knowledge on specific rare disease	^31,41,46,50-53^
	Decreased with lack of specialist communication	^41,46,52,62^
	Improved with long-term (or lifelong) patient-PCP relationship	^46-49,51,53^
	Improved with multidisciplinary care	^31^
	Comfort improved with higher volumes of rare disease patients	^43^
Health system outcomes	Encounter frequency and time taken is higher than average	^31,53,54^
	Patients, carers and providers all willing to pay for care coordination services	^28^
	Improved outcomes at population level	^31^
	Holistic care delivery	^31,54^
	Decreased health expenditure	^31^
	Specialist-only care increased unnecessary acute system demand	^43^
	Reduced diagnostic odyssey reduced whole of health system expenditure	^48,60^

Abbreviation: PCP, Primary Care Physician.

Individuals whose PCP was actively involved in their rare disease diagnosis had a shortened diagnostic delay, as compared to individuals whose PCP was not actively involved in their rare disease diagnosis.^31,43-45,48,53,55,57-60^ Diagnosis within 12 months after first suspicion of a rare disease was reported to be as high as 75% with active PCP involvement.^53^ Specifically, PCP identification of signs and symptoms of potential rare disease, as enabled through long term patient-PCP relationships and early access to care, reduced diagnostic delays.^31,58,59^ Byrne et al^55^ quantified the PCP as either diagnosing (19%) or playing a major role in diagnosis (19%) of all rare disease diagnoses in their study.

Coordinated care, with clear role delineation, reduced unplanned utilization of emergency and hospital services.^43,49,60^ A key driver of these outcomes relate to the ethos of primary care: patient- and family-centered, sustainable, longitudinal, continuous, collaborative, coordinated, integrated, and with expertise in chronic disease management and dealing with uncertainty.^28,31,41,43-55,57-59,61^ Rather than a reflecting a deficit of PCP quality of patient care, uncoordinated transfer of care to primary care resulted in increased loss-to-follow-up and reduced patient experience.^57^

For a health system, PCP involvement results in more holistic delivery of care and improved population health outcomes, particularly in shared-care models and where high encounter frequency can alert the health system to a potential new rare disease diagnosis for an individual.^31,48,60^ Despite encounter frequency and PCP time contribution being higher than average, there were overall decreased health expenditures.^31,53,54^ For example, specialist-only care increased unnecessary acute system demand for patients with Cystic Fibrosis, in comparison to when PCPs were actively involved with guideline-based screening and routine primary and preventive care for patients with Cystic Fibrosis.^43^ Patients, carers and providers were all willing to pay for care coordination services in one study.^28^ Longitudinal coordination of the patient journey between services showed benefits to patients, providers and the health system as summarized in Table 2.

Dissatisfaction was reported by PLWRD, and self-reported by PCPs, related to the perceived lack of disease-specific knowledge by the PCP.^31,41,45,46,50-53^ Improved specialist communication to PCPs, more multidisciplinary care and higher volumes of rare-disease-care may counter this.^31,41,43,46,52,62^

In relation to the quintuple aim, there were insufficient data to warrant sub-group analysis related to equity for priority populations such as those from lower socio-economic groups, poor, aged, culturally and linguistically diverse and indigenous groups, those with decreased capacities and the otherwise marginalized.^64^

### Enablers and Barriers to PCP Involvement in Rare-Disease-Care

Of the 26 papers, 20 sufficiently described implementation related information, categorized into barriers and enablers using the CFIR 2.0.^34^ These are summarized in Table 3.

**Table 3.** Enablers and Barriers for Integrated-Rare-Disease-Models-of-Care in Primary Care Settings.

Domain	Characteristic		Reference
Outer setting (wider health and social care system)	Enabler	Having a contact person for rare disease information	^41^
		All providers agree there is a role for PCPs	^49^
		Policies that mandate a named PCP	^52^
		Healthcare infrastructure to enable PCPs to provide more streamlined and integrated care	^59^
	Barrier	If transfer of care to primary care is uncoordinated, patients are lost to follow up	^57^
		Lack of specialist communication	^41,45,46,52,62^
		Fragmented system	^46,52^
		Poor change management and implementation of rare disease policies	^52^
		Not having an identified PCP	^43^
Inner setting (primary care practice)	Enabler	Funding for managing complex patients	^41^
		Rare-disease-care aligns with essential role of primary care	^28,31,41,43-46,49,50,52,54,55,57-59,61^
	Barrier	Lack of time and financial reimbursement for complex care	^51^
		Lack of information on rare diseases	^41,52,62^
Characteristics of the PCP	Enabler	Possess a broad scope of practice	^49,52,54^
		Perceive their role as care navigators, holistic approach and whole family supporters	^52^
		Comprehensive, longitudinal care	^46,47,49,51-53,58^
	Barrier	Perceived insufficient knowledge on specific rare disease	^31,41,52,53^
Integrated-rare-disease-care as an intervention	Enabler	Role clarity	^44,49,52^
		Care coordinator	^28^
		Sufficient time and reimbursement for time-intensive tasks like palliative care	^50,51,59^
		Easily accessible information and resources; clear guidelines for identification and management	^50,59^

Abbreviation: PCP, Primary Care Physician.

Complex, fragmented health systems, uncoordinated transfer of care to primary care, poor change management and lack of specialist communication were barriers to active PCP involvement in rare-disease-care.^41,43,45,46,52,57,62^ One paper described 48% of PCPs reporting to have never or almost never received a treatment summary.^51^ Structural communication options, including digital, are proposed.^28^ One study described direct transfer of care from pediatric to adult rheumatology, as compared to directing patients to contact their PCP if they developed joint symptoms, without information sharing to the PCP. This cohort were lost to follow up, and/or were reported to have poorer health outcomes.^57^ Delayed communication on medication changes and miscommunication between providers were reported.^46,52^ Two models described providing PLWRD with information, but not their PCP.^41,50^ This not sufficient for primary care contexts. Within primary care contexts, lack of rare disease information, lack of time and financial reimbursement for complex care provision were notable barriers.^41,51,52,62^

The broad scope of practice associated with the PCP role enables the provision of holistic, family-centered, comprehensive, longitudinal rare-disease-care.^43,46,47,49,51-54,58^ However, disease-specific knowledge is lacking.^31,41,52,53,63^ Shared-rare-disease-care-models were the most favorable to PCPs, in terms of their experience of providing care to PLWRD, as compared to PCP-only, or specialist-only-models.^49^ This addresses issues of fear of managing unknown conditions, lack of practical support for follow up, and overcoming long wait times for specialist care.^50,52^

## Discussion

### Outcomes of PCP Involvement in Rare-Disease-Care

Our study demonstrates that more active PCP involvement in integrated-rare-disease-care is beneficial to the health system, families, and providers. A robust primary care system improves health outcomes, efficiency, and equity.^18^ The positive role of PCPs in rare-disease-care should be strengthened with appropriate communication and referral pathways with specialists.^41,46,50,52^ Despite low prevalence of individual rare diseases, the level of PCP involvement in care is high, due to cumulative prevenance and disease complexity. Yet, real barriers to active PCP involvement exist. Such as the lack of rare-disease specific clinical guidelines, lack of referral pathways and insufficient reimbursement for the time needed to provide complex family-centered care. Comparatively, easily actionable barriers included lack of communication from specialists and information technology infrastructure for telemedicine. Communication can be improved through fostering links and relationships with hospital based specialists,^52^ co-location,^43^ shared care plans,^28,43,48,51^ sharing results,^44^ and/or a care-coordinator/navigator.^28^ The care-coordinator role, their scope of practice, frequency of contact, mode of communication, location require careful planning and co-design with PLWRD and PCPs.^28^

This review highlights that PCPs have a unique advantage in providing long-term health care, due to their considerable expertise in managing multi-system disease, broad scope of practice, bio-psycho-social approach and confidence in dealing with uncertainty.^31,44-49,51-55,58-60,65^ Author de Vries posits that PCPs, as the health system gatekeeper, may create diagnostic delays, yet integrating the PCPs role in rare disease care is the most equitable and efficient way to structure a universally accessible health system that balances budget expenditure with patient and provider needs and experience.^40,46^ Primary care models of rare disease care can significantly improve health system performance through holistic care delivery and simultaneous decrease health care expenditure.^31^ This is unsurprising given primary care system’s role in coordinating local and regional networks, integrating care, addressing local population needs, providing preventive, holistic care and health promotion and education.^31^

Insufficient data were available to undertake subgroup analysis in relation to equity. However, Morris et al^28^ provide hypothetical models of care which take rurality into account. Co-design of optimal integrated-rare-disease-models-of-care that support all priority populations should be an important avenue of future research.

### Enablers and Barriers to PCP Involvement in Rare-Disease-Care

Traditional quality improvement approaches focus on increasing awareness and education on rare diseases.^15,21,66^ Given there are more than 9000 individual rare diseases, it is unrealistic to focus on building the knowledge of PCPs on the intricacies of every specific condition.^29,47,48,66^ This review highlights the need to focus on practical guidance and tools, appropriate reimbursement, access to specialist services and referral and communication pathways, rather than PCP knowledge alone in order to reap the benefits of the key role of PCPs in integrated-rare-disease-care.^41,46,50,52^ PCPs provide comprehensive, coordinated, continuous, accessible and family-centered care to people across the lifespan.^67^ In addition to current primary health system governance, specific enablers for improved rare disease care such as sufficient time and reimbursement for complex care delivery, improved interface between PCP and specialist providers with greater access to disease-specific knowledge and information, clear role delineation and shared care models to support the PCP are needed.^31,41,46,49-55,57,59,60,62^

Integrated-rare-disease-care models in this review ranged from specialist-only to full shared-care models, with evidence supporting the overall superiority of shared-care models. Enablers for development of an integrated-rare-disease-model-of-care for primary care included role clarity, sufficient time and reimbursement for complex care, accessible and clear information, and a dedicated care coordinator.^28,44,49-52,59^ Enablers for implementation of integrated-rare-disease-care include: national rare disease strategies, technical enablers for improved communication with specialists, adequate reimbursement for time and availability of a time-limited care coordinator.

Our review supports the critical need to include PCP perspectives in the design of an integrated-rare-disease-model-of-care. Additionally, future models of care should explore distinctions between provider and patient preferences. In this scoping review, providers reported a preference for all care to be coordinated by the Care Coordinator, whereas families preferred to coordinate their own care but for the Care Coordinator to play a supportive role, when required.^28^

### Strengths and Limitations

Rare disease advocacy groups are often charities without the funds to publish academic literature but raise valuable input for models of care design related to this population group. Grey literature search was attempted, yet none were found to sufficiently relate to family practice settings for inclusion in this review. To identify as many studies as possible, we conducted a comprehensive search inclusive of all primary health care settings, later refining to family practice or equivalent. Despite our inclusive strategy, we are unlikely to have captured every relevant paper.

A limitation of applying the CFIR 2.0 to understand pre-implementation for an integrated-rare-disease-model-of-care in primary care is that the CFIR 2.0 does not make consideration of patient characteristics, preferences, capacity, and capability. This is an important element in the care of PLWRD. Morris et al’s^28^ hypothetical models of care have considered family perspectives.

## Conclusions

Any new models of care should be developed by, or with PCPs. Exploration of PCP perspectives within their workplace and broader health system is warranted. Despite the dwindling PCP workforce, an effort needs to be made by the research community to involve end-users in research.

Future models of care should articulate role clarity, improved communication, the need for someone to take responsibility for organizing care delivery. These findings may provide support for various international policy initiatives.^24,25^ With the World Health Organization and United Nations advocating for rare disease centers of excellence, PCPs can play a significant role.

There were insufficient findings in the current review to provide information on how rare-disease-care is organized and delivered between primary care and the Social Care sector. Whole-family, supportive care is particularly needed for these complex and often progressive diseases.^20,24,25^ This is a significant gap in literature and deserves further inquiry. Despite this, or perhaps because of it, this research is also transferrable to health systems in low-to-middle income countries which may be planning to address the fragmentation of care delivered to PLWRD.

## Supplemental Material

sj-zip-1-jpc-10.1177_21501319241311567 – Supplemental material for Integrated Care for People Living With Rare Disease: A Scoping Review on Primary Care Models in Organization for Economic Cooperation and Development Countries Supplemental material, sj-zip-1-jpc-10.1177_21501319241311567 for Integrated Care for People Living With Rare Disease: A Scoping Review on Primary Care Models in Organization for Economic Cooperation and Development Countries by Nada Vidic, Anna McGlynn, Fatemeh Abdi, Chun Wah Michael Tam, Reginald Michael Crampton, Kean-Seng Lim, Elizabeth Emma Palmer, Natalie Taylor and Ben Harris-Roxas in Journal of Primary Care & Community Health
