# Supplementary material for: Integrated Care for People Living With Rare Disease: A Scoping Review on Primary Care Models in Organization for Economic Cooperation and Development Countries
Source: J Prim Care Community Health. 2025 Jan 8;16:21501319241311567. doi: 10.1177/21501319241311567 (PMC11707790; doi:10.1177/21501319241311567)
Supplement: sj-zip-1-jpc-10.1177_21501319241311567 – Supplemental material for Integrated Care for People Living With Rare Disease: A Scoping Review on Primary Care Models in Organization for Economic Cooperation and Development Countries [file sj-zip-1-jpc-10.1177_21501319241311567.zip › Supplement VIII Consensus Reporting Items for Studies in Primary Care (CRISP) reporting item Checklist.pdf]

Figure 1. Consensus Reporting Items for Studies in Primary Care reporting item Checklist and Instructions.

Reporting Item Checklist

No.	Reporting Item[a] (See instructions below. Not all items apply to all study designs.)	Included[b] Y	N	NA	Section[c]	Notes[d]
**1.**	**Include "primary care" and/or discipline-specific terms in the title, abstract, and/or key words.**	☒	☐	☐	I	
**2.**	**Describe the study rationale and importance for primary care.**					
2a.	Explain the rationale for the research question and how it relates to primary care.	☒	☐	☐	I	
2b.	Describe the importance or relevance of the topic under study in the primary care setting.	☒	☐	☐	I	
2c.	Identify any theory, model, or framework used, and explain why it is appropriate to the research question in primary care.	☒	☐	☐	I	
**3.**	**Describe the research team's primary care experience and collaboration.**					
3a.	Describe the research team's expertise and experience in primary care practice and/or research.	☒	☐	☐	M	
3b.	Describe whether and how primary care patients, practicing clinicians, community members, or other stakeholders were involved in the research process.	☒	☐	☐	M	
**4.**	**Describe the study participants and populations in the context of primary care.**					
4a.	Use person-focused language to refer to the research populations and participants, or use terms based on patient preferences.	☒	☐	☐	R	
4b.	If reporting personal characteristics of participants, report the source of the data, the rationale for using it, and the rationale for any classifications used.	☐	☐	☒	R	
4c.	Describe the participants and populations in sufficient detail to allow comparison to other primary care patient populations.	☒	☐	☐	R	
4d.	Specify whether participants have preexisting therapeutic relationships with the clinical team or are new patients.	☐	☐	☒	M, R	Reporting on this varied between studies
**5.**	**Describe the conditions under study in the context of primary care.**					
5a.	Describe whether the condition under study is acute or chronic.	☒	☐	☐	M, R	
5b.	Report how multimorbidity is considered and how it might affect interpretation of the study findings/results.	☒	☐	☐	M	Treatment of concurrent illnesses noted in R, D
**6.**	**Describe the clinical encounter under study in the context of primary care**					
6a.	Specify whether the study focus is an isolated clinical encounter or a longitudinal course of care. If it is an isolated clinical encounter, specify whether it is the first visit or a follow-up visit for the condition under study.	☒	☐	☐	M	Scoping review, N/A for methods<br>Noted in R, D
**7.**	**Describe the patient care team.**					
7a.	If care is delivered by teams, describe the team members and their roles.	☒	☐	☐	R	
7b.	For each clinician category, report profession, specialty, and qualifications.	☒	☐	☐	R	
**8.**	**Describe the study interventions in the context of primary care.**					
8a.	Describe interventions and their implementation in sufficient detail to enable the reader to assess applicability in their own setting.	☒	☐	☐	M	Scoping review, N/A for methods<br>Noted in R, D
8b.	Describe any clustering or grouping of patients, participants, clinicians, teams, or practices, and how it was addressed in the analysis.	☒	☐	☐	M, R	
8c.	Describe the health care system in sufficient detail to allow comparisons to other systems.	☒	☐	☐	I, D	
**9.**	**Describe study measures used and their relevance to primary care.**					
9a.	Report whether study measurement tools have been validated in primary care populations or settings.	☐	☐	☒	M	Scoping review methods, relevant to all discuiplines
9b.	Describe how the measurement tools used are meaningful to primary care patients and their care.	☐	☒	☐	M	Noted in I, D
9c.	Report findings/results to be clinically interpretable by primary care clinicians and patients.	☐	☐	☒	R	Scoping review
**10.**	**Discuss the meaning of study findings/results in the context of primary care.**					
10a.	Discuss implications of the study findings/results for research, patient care, education, and policy with specific focus on primary care.	☒	☐	☐	D	
10b.	Discuss the implications of study recommendations on demands and priorities in primary care practice.	☒	☐	☐	D	
10c.	Comment on any research processes that might influence the applicability of the study findings/results in diverse primary care settings.	☒	☐	☐	D	

CRISP = Consensus Reporting Items for Studies in Primary Care; D = discussion; I = introduction; M = methods; N = no; NA = not applicable, R = results; Y = yes.

Instructions: (1) The CRISP Checklist aids researchers in meeting readers' needs by including content that our primary care research community feels is important for the validity, quality, and usefulness of primary care research reports. Authors and editors make final decisions. (2) Primary care research involves a wide variety of methods, study designs, topics, and settings; thus, not all items apply to all studies. Please respond to each item but note if it is not applicable for your study. If an item is missing from your report but applies to your study, simply note that and provide some brief explanation of why it is not included. (3) Authors should also use other reporting guidelines appropriate for their study and report. Some CRISP items may overlap with other guidelines. Version 1.0, published: October 4, 2023, CRISP (https://crisp-pc.org/).

[a] For more information plus explanation, and examples of each item, see the Supplemental Appendix.
[b] Indicate whether the item is included in your report: yes, no, or not applicable. If the item applies to the study design but is not included in the report, please provide an explanation.
[c] Suggested location for the item in research reports according to the IMRaD (Introduction, Methods, Results, and Discussion) format.
[d] Notes on the location of the item in your report (by line, page, or section) or reason for omission of the item from the report.
