# Supplementary material for: Integrated Care for People Living With Rare Disease: A Scoping Review on Primary Care Models in Organization for Economic Cooperation and Development Countries
Source: J Prim Care Community Health. 2025 Jan 8;16:21501319241311567. doi: 10.1177/21501319241311567 (PMC11707790; doi:10.1177/21501319241311567)
Supplement: sj-zip-1-jpc-10.1177_21501319241311567 – Supplemental material for Integrated Care for People Living With Rare Disease: A Scoping Review on Primary Care Models in Organization for Economic Cooperation and Development Countries [file sj-zip-1-jpc-10.1177_21501319241311567.zip › Supplement IV Descriptive Characteristics of Included Studies.docx]

Supplement IV: Descriptive Characteristics of Included Studies

| **Descriptive characteristics** | | |
| --- | --- | --- |
| **Type of Publication** | **Number** | **References** |
| Peer reviewed (Original Research) | 12 | [Baqué, 2019] [Boffin, 2018] [Buendia, 2022] [Byrne, 2020] [Carroll, 2021] [Clayton-Smith, 2019] [Druschke, 2021] [Jo, 2019] [McClain, 2014] [Mikola, 2022] [Morris, 2022] [Willis, 2015] |
| Peer reviewed (Review) | 3 | [Birnkrant, 2018] [Ferreira, 2023] [Schraeder, 2022] |
| Peer reviewed (Guidance Document) | 4 | [Carls, 2017] [Evans, 2021] [Noritz, 2018] [Palmer, 2023] |
| Peer reviewed (Commentary or Opinion) | 2 | [Auth, 2023] [de Vries, 2018] |
| Peer reviewed (Editorial) | 3 | [Dudding-Byth, 2015] [Evans, 2016] [Lewis, 2015] |
| Peer reviewed (Protocol) | 1 | [Schraeder, 2021] |
| Grey Literature (Pre-print) | 1 | [McMullan, 2021] |
| **Country or Region (of study)** | **Number** | **References** |
| America | 7 | [Auth, 2023] [Birnkrant, 2018] [Carls, 2017] [Jo, 2019] [Lewis, 2015] [McClain, 2014] [Noritz, 2018] |
| Australia | 2 | [Dudding-Byth, 2015] [Palmer, 2023] |
| Belgium | 1 | [Boffin, 2018] |
| Brazil | 1 | [Ferreira, 2023] |
| Canada | 3 | [Carroll, 2021] [Schraeder, 2021] [Schraeder, 2022] |
| European Union | 1 | [Clayton-Smith, 2019] |
| England | 5 | [Buendia, 2022] [Evans, 2016] [Evans, 2021] [Morris, 2022] [Willis, 2015] |
| Finland | 1 | [Mikola, 2022] |
| France | 1 | [Baqué, 2019] |
| Germany | 1 | [Druschke, 2021] |
| Ireland | 2 | [Byrne, 2020] [McMullan, 2021] |
| Netherlands | 1 | [Baqué, 2019] [Boffin, 2018] [Buendia, 2022] [Byrne, 2020] [Carroll, 2021] [Clayton-Smith, 2019] [Druschke, 2021] [Jo, 2019] [McClain, 2014] [Mikola, 2022] [Morris, 2022] [Willis, 2015] |
