# Supplementary material for: Integrated Care for People Living With Rare Disease: A Scoping Review on Primary Care Models in Organization for Economic Cooperation and Development Countries
Source: J Prim Care Community Health. 2025 Jan 8;16:21501319241311567. doi: 10.1177/21501319241311567 (PMC11707790; doi:10.1177/21501319241311567)
Supplement: sj-zip-1-jpc-10.1177_21501319241311567 – Supplemental material for Integrated Care for People Living With Rare Disease: A Scoping Review on Primary Care Models in Organization for Economic Cooperation and Development Countries [file sj-zip-1-jpc-10.1177_21501319241311567.zip › Supplement V Study Characteristics Disease, Context and Care Delivery.docx]

| **Study Characteristics: Disease, Context and Care Delivery** | | |
| --- | --- | --- |
| **Participant: Named Rare Disease** | **Number** | **References** |
| Cystic Fibrosis | 5 | [Auth, 2023], [Carroll, 2021], [Lewis, 2015], [Schraeder, 2021], [Willis, 2016] |
| Duchenne Muscular Dystrophy | 3 | [Birnkrant, 2018], [Carls, 2017], [Noritz, 2018] |
| Foeatal Valporate Spectrum Disorder | 1 | [Clayton-Smith, 2019] |
| Juvenile Idiopathic Arthritis | 1 | [Mikola, 2022] |
| Rare Disease Agnostic (or numerous mentioned) | 15 | [Boffin, 2018], [Buendia, 2022], [Byrne, 2020], [de Vries, 2018], [Druschke, 2021], [Dudding-Byth, 2015], [Evans, 2016], [Evans, 2021], [Ferreira, 2023], [Jo, 2019], [McClain, 2014], [McMullan, 2021], [Morris, 2022], [Schraeder, 2022], [Palmer, 2023] |
| Rare Skin Diseases | 1 | [Baqué, 2019] |
| **Context** | **Number** | **References** |
| General Practice / Family Practice | 24 | [Baqué, 2019], [Birnkrant, 2018]~#, [Boffin, 2018], [Buendia, 2022]~, [Byrne, 2020], [Carroll, 2021], [Clayton-Smith, 2019], [de Vries, 2018], [Druschke, 2021]#, [Dudding-Byth, 2015], [Evans, 2016], [Evans, 2021], [Ferreira, 2023], [Jo, 2019]#, [Lewis, 2015], [McClain, 2014]#, [McMullan, 2021], [Mikola, 2022]#, [Morris, 2022], [Noritz, 2018]#, [Schraeder, 2021]~, [Schraeder, 2022, [Palmer, 2023], [Willis, 2016] |
| Patient-Centred Medical Home | 2 | [Auth, 2023]#, [Carls, 2017]# |
| Note: ~Equivalent models of primary care such as general practice. #Primary care *and* another setting. | | |
| **Care Delivery** | **Number** | **References** |
| Describes how rare disease care is currently organised and delivered, along the continuum of care | 7 | [Birnkrant, 2018], [Carls, 2017], [Clayton-Smith, 2019], [Druschke, 2021], [Noritz, 2018], [Schraeder, 2021], [Schraeder, 2022] |
| Evaluates how rare disease care is currently organised and delivered, along the continuum of care | 16 | [Baqué, 2019], [Boffin, 2018], [Buendia, 2022]#, [Byrne, 2020], [Carroll, 2021], [de Vries, 2018], [Dudding-Byth, 2015]^, [Evans, 2016]^, [Ferreira, 2023]^, [Jo, 2019], [Lewis, 2015], [McClain, 2014], [McMullan, 2021], [Mikola, 2022], [Morris, 2022]*, [Willis, 2016] |
| Investigates /proposes ideal rare disease model of care delivery | 4 | [Auth, 2023], [Evans, 2021], [Morris, 2022]*, [Palmer, 2023] |
| Note: # Evaluates implementation of an intervention to how rare disease care is currently organised and delivered, along the continuum of care. ^Focused on the role of the family physician. *Relates to more than one concept. ~Protocol paper | | |
