# Supplementary material for: Integrated Care for People Living With Rare Disease: A Scoping Review on Primary Care Models in Organization for Economic Cooperation and Development Countries
Source: J Prim Care Community Health. 2025 Jan 8;16:21501319241311567. doi: 10.1177/21501319241311567 (PMC11707790; doi:10.1177/21501319241311567)
Supplement: sj-zip-1-jpc-10.1177_21501319241311567 – Supplemental material for Integrated Care for People Living With Rare Disease: A Scoping Review on Primary Care Models in Organization for Economic Cooperation and Development Countries [file sj-zip-1-jpc-10.1177_21501319241311567.zip › Supplement VI Main Characteristics of Included Studies.docx]

|  | **Study ID** | **Journal** | **Study Design** | **Aim or major objective** | **Phenomenon of Interest** |
| --- | --- | --- | --- | --- | --- |
| **Original Research** | Baqué, 2019 | La Presse Medicale | Survey / Questionnaire | To identify rare skin diseases in a reference centre, to describe the difficulties encountered by general practitioners (GPs) in management of these uncommon cases, and to pinpoint the characteristics of the GPs having the most problems | What do GPs have most problems with regarding management of rare skin diseases? What are the characteristic s of the GPs experiencing most problems (socio-economic, geographical, gender, years of experience)? |
|  | Boffin, 2018 | International Journal of Environmental Research and Public Health | Survey / Questionnaire | To examine care characteristics of rare disease patients; and to examine the importance of rare diseases in general practice by its caseload (number of cases, estimated prevalence, and age differentiation between sentinel general practices and Belgian general practice population) | Caseload of rare disease in general practice; encounter frequency of people living with rare disease in general practice |
|  | Buendia, 2022 | Orphanet Journal of Rare Diseases | Pilot feasibility study | Pilot study to test implementability (specifically feasibility) of an IT-enabled population level rare disease case finding tool in primary care | Feasibility of a digitally enabled case finding tool in primary care |
|  | Byrne, 2020 | Irish Journal of Medical Science | Survey/ Questionnaire | To estimate the general practice clinical workload attributable to selected rare diseases and assess the use of relevant information sources | The role of primary care (GP) in the Irish rare disease patient care journey. Clinical workload attributed to rare disease (frequency of consultations, and referrals to specialists. GP's self-reported role in case and management of the patient), and use of relevant information sources by GPs |
|  | Carroll, 2021 | Canadian Family Physician | Survey / Questionnaire | To explore primary care providers’ preferred roles and confidence in (i) caring for infants receiving a positive cystic fibrosis newborn screening result; and (ii) management of cystic fibrosis family planning issues | Primary care providers’ preferred roles in providing well-baby care for infants with positive cystic fibrosis screening results |
|  | Clayton-Smith, 2019 | Orphanet Journal of Rare Diseases | Guidance document (AGREE II) | To provide guidance on the most effective management of the medical and neurodevelopmental problems of individuals demonstrating the effects of prenatal exposure to valproic acid or sodium valproate from infancy to adulthood | Diagnosis and management of Foetal Valproate Spectrum Disorder; prevention of in utero exposure, consensus statement |
|  | Druschke, 2021 | Orphanet Journal of Rare Diseases | Mixed methods: Survey / Questionnaire and Interview | To understand the preferences, expectations and needs of general practitioners and paediatricians for the care of patients with rare diseases (i) knowledge about the centre for rare disease; (ii) satisfaction with services provided by the centre for rare diseases and (iii) expectations and needs. | The interface management between primary care physicians and the centres for rare diseases |
|  | Jo, 2019 | British Medical Journal Open | Cross sectional study (national representative dataset) | To explore characteristics of visits for patients with rare diseases seen by primary care physicians | The prevalence of rare diseases in primary care visits |
|  | McClain, 2014 | Clinical Paediatrics | Survey / Questionnaire | To assess primary care pediatric providers’ comfort with co-managing patients with rare conditions, and the quality of communication from specialists and preferences regarding communication and role delineation | Co-management of patients with rare conditions: specialists provide diagnostic confirmation and rapid initiation of management. "*The process of engaging specialists in the management of the chronic condition, while necessary, might separate care of that condition from other aspects of the child’s health care (preventive care, acute illness care) and pose the risk of fragmentation of care between the specialists and the child’s primary care medical home.*" p1 |
|  | Mikola, 2022 | Scandinavian Journal of Rheumatology | Interview (longitudinal cohort) | To analyse the timing of the patients’ transition to adult care, and patients’ self-management skills with the process and the quality of the transition | Transition from paediatric to adult care |
|  | Morris, 2022 | Health and Social Care Delivery Research | Mixed methods: scoping review, qualitative interview, survey, discrete choice experiment | To investigate if and how care of people with rare conditions is co-ordinated in the United Kingdom, and how people affected by rare conditions would like care to be co-ordinated | Care coordination |
|  | Willis, 2015 | British Journal of General Practice | Interview | To explore GPs and practice-nurses’ perspectives about the role of primary care in supporting children (and their families) with long term conditions, including cystic fibrosis | Primary care practitioner's views and experiences of supporting children with long term conditions, including cystic fibrosis |
| **Review Article** | Birnkrant, 2018 | The Lancet, Neurology | Review | To provide a guide to the acute and chronic medical conditions that first-line providers are likely to encounter. Inclusive of assessments and interventions that are designed to improve mental health and independence, functionality, and quality of life in critical domains of living, including health care, education, employment, interpersonal relationships, and intimacy | The role of primary care (and emergency) management of acute and chronic aspects of Duchenne Muscular Dystrophy. Including psychosocial issues and transitions of care, due to the unique set of challenges relating to prolonged survival in the cohort |
|  | Ferreira, 2023 | Journal of Global Health | Scoping Review, systematic review (both terms used) | To collate available evidence of the impact of primary health care on patients with rare disease and summarise published information from multiple stakeholders about the perceived usefulness and barriers to effective use of the primary health care system | The role of the primary healthcare system in rare disease care; the effectiveness of primary healthcare system approach on managing patients with rare diseases; the practice and structural gaps in the care network that hinders integrated rare disease care |
|  | Schraeder, 2022 | Journal of Primary Care & Community Health | Scoping Review | To summarise how primary care providers (such as family physicians) support adolescents and young adults with chronic conditions, including cystic fibrosis, transitioning from pediatric- to adult- specialty care | Primary care visits pre- and post- transition; benefits and challenges of primary health provider involvement in transition; and models of collaborative primary-specialty care models currently exist for transition |
| **Guidance Document** | Carls, 2017 | The Journal of the American Osteopathic Association | Guidance document | To provide osteopathic primary care physicians with current and relevant information regarding Duchenne Muscular Dystrophy diagnosis and management | The role of primary care physicians in expediting diagnosis and treatment coordination |
|  | Evans, 2021 | InnovAiT | Guidance Document | Nil stated. To inform clinicians on how to identify, manage and support patients with a rare disease | The role of the GP in rare disease identification, management and support of patients and their families |
|  | Noritz, 2018 | Pediatrics | Guidance document | To provide guidance to primary care physicians and emergency physicians to skilfully care for patients with Duchenne's Muscular Dystrophy in their respective settings, optimising patient outcomes | Care management: Applying general principles of primary care, such as timely immunisations, anticipatory safety counselling, behavioural screening, routine nutritional and developmental assessments. Coordination with medical specialists. Role of trusted advisor regarding medical decisions, psychological, behavioural and educational domains. Emergency care planning. Monitoring, recognising and acting on specific medical issues that arise in patients with Duchenne's Muscular Dystrophy |
|  | Palmer, 2023 | Medicine Today | Guidance document | To highlight common challenges of rare disease, new approaches and resources that have been developed to help doctors in Australia address priorities in the Australian rare disease strategic action plan | The common challenges of rare disease, noting resources and new developments for Australian doctors |
| **Commentary, Opinion or Editorial** | Auth, 2023 | Journal of Primary Care and Community Health | Commentary | To provide relevant background on diagnosing and treating cystic fibrosis, current preventative screening measures, and potential models for delivering primary care to adults with cystic fibrosis | Integrating primary care into the management of cystic fibrosis, via a co-located shared model of care |
|  | de Vries, 2018 | British Journal of General Practice | Debate and analysis, with embedded qualitative interview | To reflect on both organisational and GP factors that contribute to the problem of gatekeeping, and offer suggestions on organisational and educational approaches to tackling it | Organisational and GP factors that cause delays in referral; possibly resulting in permanent organ damage, reduction in quality of life, and increased healthcare costs |
|  | Dudding-Byth, 2015 | Australian Family Physician | Editorial | To outline the challenges faced by the rare disease community, and the role of the primary care physician to advocate for answers as their patients transition through the healthcare system | The role of the family physician in rare disease, with the emergence of the 'expert patient' and shift in the traditional doctor patient interaction. The patient who seeks a collaborative and empowering relationship with their physician, followed by referral to relevant specialist based on differential diagnoses |
|  | Evans, 2016 | British Journal of General Practice | Editorial | Nil stated. To inform primary care physicians on how to recognise rare disease | The role of the family physician in rare disease, with the emergence of the 'expert patient' and shift in the traditional doctor patient interaction |
|  | Lewis, 2015 | American Family Physician | Editorial | Nil stated. To inform family physicians on how they can make valuable contributions to the care of patients with cystic fibrosis reaching adulthood | The role of the family physician in the management of cystic fibrosis; management of body mass index, screening and management of cystic fibrosis-related diabetes mellitus; promoting general health including exercise; encouraging medication and therapy compliance. Preventative care such as that for the general population (cancer screening, routine immunisations. Quarterly review by cystic fibrosis clinic multidisciplinary team (does not include a GP) |
| **Protocol or Pre-Print** | McMullan, 2021 | medRxiv preprint | Survey / Questionnaire | To gain an understanding of GP’s perception and experience of rare diseases | GP’s perception and experience of rare diseases. Specifically, GPs career to date, interactions with rare disease patients, challenges faced, the exchange of information with patients, priorities for the future, support for such patients and training |
|  | Schraeder, 2021 | Systematic Reviews | Protocol: systematic review | A protocol for a systematic review with the aim to synthesise the available literature on the roles of primary care providers (such as family physicians) for adolescents and young adults with chronic conditions, including cystic fibrosis, leaving pediatric specialty care and identify potential benefits and challenges of maintaining primary care provider involvement during transition into adult services | Role of the primary care provider (such as family physician) in transition from paediatrics to adult care services for adolescents and young adults with chronic conditions (including cystic fibrosis) |
