# Supplementary material for: Integrated Care for People Living With Rare Disease: A Scoping Review on Primary Care Models in Organization for Economic Cooperation and Development Countries
Source: J Prim Care Community Health. 2025 Jan 8;16:21501319241311567. doi: 10.1177/21501319241311567 (PMC11707790; doi:10.1177/21501319241311567)
Supplement: sj-zip-1-jpc-10.1177_21501319241311567 – Supplemental material for Integrated Care for People Living With Rare Disease: A Scoping Review on Primary Care Models in Organization for Economic Cooperation and Development Countries [file sj-zip-1-jpc-10.1177_21501319241311567.zip › Supplement II Data extraction instrument .docx]

# Supplement II: Data extraction instrument

| Reviewer |  | Date |  |
| --- | --- | --- | --- |

Article information:

| Author |  | Year |  |
| --- | --- | --- | --- |
| Journal |  | Record Number |  |
| Country |  |  |  |

Study Description:

| Methodological / Study design |  |
| --- | --- |
| Method |  |
| Phenomena of interest |  |
| Setting |  |
| Geographical |  |
| Cultural |  |
| Participants |  |
| Disease described (if relevant) |  |
| Data analysis |  |
| Author conclusions |  |
| Comments |  |

Model of Care:

| The model of care / Component of care delivery |  |
| --- | --- |
| Inner setting description (where care is delivered) |  |
| Outer setting description (Context within which inner setting sits) |  |
| Characteristics of the individual/s delivering care (profession, other particulars) |  |
| Care delivery process |  |
| Care delivery outcomes |  |
| Factors related to feasibility in general practice |  |
| Factors related to sustainability in general practice |  |

| Key Findings | Illustration from publication (page number) | Evidence | | |
| --- | --- | --- | --- | --- |
|  |  | Unequivocal | Credible | Unsupported |
|  |  |  |  |  |
|  |  |  |  |  |

Extraction of findings complete: Yes / No
