# Supplementary material for: Integrated Care for People Living With Rare Disease: A Scoping Review on Primary Care Models in Organization for Economic Cooperation and Development Countries
Source: J Prim Care Community Health. 2025 Jan 8;16:21501319241311567. doi: 10.1177/21501319241311567 (PMC11707790; doi:10.1177/21501319241311567)
Supplement: sj-zip-1-jpc-10.1177_21501319241311567 – Supplemental material for Integrated Care for People Living With Rare Disease: A Scoping Review on Primary Care Models in Organization for Economic Cooperation and Development Countries [file sj-zip-1-jpc-10.1177_21501319241311567.zip › Supplement I Literature Eligibility Criteria Clean.docx]

# Supplement I: Literature Eligibility Criteria

| **Theme** | **Inclusion** | **Exclusion** |
| --- | --- | --- |
| Participants | Diseases and conditions with a prevalence of lower than 1 in 2000^a^ | Chronic or complex disease without rarity |
|  | The collective words of “rare” or “orphan” disease | Diseases and conditions not affecting humans |
|  | Literature addressing the research question while using a specific rare^b^ disease as an exemplar |  |
|  | Undiagnosed^c^ diseases |  |
|  | Paediatric, adult, and aged populations |  |
|  | All geographical areas |  |
|  | All cultural backgrounds |  |
| Concept | Literature describing and/or testing at least one component of how care is organised or delivered, across care settings | Literature unrelated to health service delivery. Such as studies focused on gene discovery, orphan-drugs, or medication efficacy testing |
|  | Relating to all aspects of care; diagnosis, continuous treatment, and management | Singular aspect of consultations. Such as pre-conception screening or diagnostic tests |
|  | Includes integrated rare disease care delivery components such as:    Process: person centredness, patient/family centredness, comprehensiveness, coordinated, continuous, referrals and referral pathways, service delivery payment processes, web-based/paper-based.    Structure: models of care, management plans. Includes enablers of integrated rare disease care delivery such as information sharing platforms, funding, reporting, governing structures    Outcome: all findings in relation to care outcomes, health outcomes, quality of life, disability adjusted life years, relating to how care is organised and delivered | Acute or episodic care |
|  |  |  |
|  |  |  |
|  |  |  |
| Context | General Practice | Acute care settings |
|  | Family Practice | Aboriginal health services |
|  |  | Community health services |
|  |  | Residential aged care facilities |
|  |  | Private specialists |
|  |  | Clinical research studies that also describe components of integrated rare disease care, along the care continuum |
| Other |  | Not in the date range (2013-2023)^d^ |
|  |  | Not in OECD countries^e^ |
|  |  | Not in English language |

^a^As defined by OrphaNET; ^b^A list of specific rare diseases used in research as a representative sample can be viewed in Appendix I; ^c^A term is used in the rare disease field where health care providers may assess that a person’s phenotype strongly suggests the presence of a genetic condition. However, a diagnosis that explains all symptoms is unable to be made. This may be because the condition has not yet been discovered (thus a diagnosis is not yet possible) or because the patient is still undergoing diagnostic pathways and a correct diagnosis has not yet been made; ^d^Limiter designed to include only contemporary models of integrated rare disease care delivery in the general practice setting, and most recent developments in the rare disease sector; ^e^Organizations for Economic Co-operation and Development.
